# Efficacy of Different Irrigation Solutions on Bacterial Biofilm in Periprosthetic Joint Infections: A Systematic Review and Network Meta-Analysis †

**DOI:** 10.3390/life15040568

**Published:** 2025-04-01

**Authors:** Marcos González-Alonso, Adrián Guerra-González, Vega Villar-Suárez, Belén Fernández-Castilla, Jaime A. Sánchez-Lázaro

**Affiliations:** 1 Orthopaedic Surgery Department, Complejo Asistencial Universitario de León, 24008 León, Spain; 2 University of León-Universidad de León (IBIOMED), 24007 León, Spain; 3 Orthopaedic Surgery Department, Hospital of El Bierzo, 24404 Ponferrada, Spain; 4 Department of Methodology of Behavioral Sciences, Faculty of Psychology, National University of Distance Education, 28015 Madrid, Spain

**Keywords:** periprosthetic joint infection, irrigation, biofilm, antiseptic, povidone–iodine, bactisure

## Abstract

**Background**: Chemical debridement with an irrigating solution associated with surgical debridement has proven to be useful in the treatment of periprosthetic joint infection (PJI). The aim of the study was to perform a systematic review and meta-analysis of the current literature regarding the efficacy of different irrigation solutions on bacterial biofilm. **Methods**: This study was conducted according to the Preferred Reporting Items for Systematic reviews and Meta-Analysis extension for Network Meta-Analysis (PRISMA-NM) checklist. A Network Meta-Analysis was performed to analyze which irrigation solution achieved a greater reduction in colony-forming units (CFU) after specific exposure times in vitro. Effect size was measured using the log ratio of means (logRoM) and 95% confidence intervals (95% CI). The rank probability for each treatment was calculated using P-values. **Results**: After discarding duplicates, screening, and reviewing the full texts, four studies with 10 irrigation solutions for different exposure durations were included. The solutions were studied on mature biofilms of the most frequent bacteria. The greatest effect was achieved with 10% povidone–iodine for 5 min (est.: −12.02; 95% CI: −14.04, −9.99). The best-ranked solutions were 10% povidone–iodine for 5, 3, and 1 min (respective *p*-values: 0.977, 0.932, 0.887), and its combination with hydrogen peroxide for 3 min (*p*-score: 0.836). Povidone–iodine 0.3% for 5 min completed the top five ranked solutions in this study (*p*-score: 0.761). **Conclusions**: Our results show that 10% povidone–iodine is the best antiseptic solution when studied in vitro in the context of prosthetic joint infection. Further research in these areas is necessary to determine whether these results are reproducible with in vivo situations.

## 1. Introduction

Periprosthetic joint infections (PJIs) represent a significant complication in orthopedic surgery, leading to increased morbidity, prolonged hospital stays, and substantial healthcare costs [1,2]. Currently, 14.8% of hip arthroplasty revisions and 25% of knee arthroplasty revisions are due to infections [3]. The management of PJIs is particularly challenging due to the formation of bacterial biofilms on implant surfaces, which confer resistance to antimicrobial treatments and host immune defenses, significantly complicating eradication efforts. Management of early or acute PJI typically involves debridement, antibiotics, irrigation, and implant retention [4]. This variability highlights the ongoing debate regarding optimal intraoperative strategies to enhance bacterial clearance and reduce treatment failures. However, outcomes remain inconsistent, with some studies reporting failure rates as high as 84% [5]. These biofilms confer resistance to both antimicrobial therapies and host immune responses, complicating eradication efforts [6].

While isotonic saline solution (0.9% sodium chloride) has historically served as the standard irrigant in clinical practice, contemporary surgical protocols are increasingly incorporating adjunctive agents into irrigation solutions [7]. This evolving approach includes the integration of surfactant compounds (detergents), antimicrobial agents, and antiseptic formulations as components of updated therapeutic strategies [8]. Various antiseptic solutions are utilized intraoperatively to mitigate biofilm formation and manage PJIs. Commonly used agents include povidone–iodine (PI), chlorhexidine gluconate, acetic acid, and hydrogen peroxide. There are also commercially available pre-mixed irrigation solutions that have been developed to incorporate adjuvant compounds, including surfactant agents, broad-spectrum antimicrobials, and antiseptic components, as part of their combined-action therapeutic design. However, the literature presents conflicting evidence regarding their relative efficacy and optimal usage parameters [9,10]. Notably, the majority of scientific investigations regarding irrigation protocols emphasize prophylactic applications against PJI [11,12], while high-quality evidence addressing curative interventions for confirmed PJI cases and longitudinal recurrence metrics remains comparatively underdeveloped in the current literature. For instance, while some studies suggest superior biofilm eradication with certain solutions, others report minimal differences or raise concerns about cytotoxicity to surrounding tissues [13,14,15].

Irrigation may help combat bacterial adhesion. However, despite numerous studies, a consensus on the most effective antiseptic solution for biofilm eradication in PJIs remains elusive. This lack of agreement may stem from variations in study designs, bacterial strains tested, and assessment methodologies. Consequently, there is a critical need for a comprehensive analysis that synthesizes existing data to provide clearer guidance for clinical practice.

Given these discrepancies, a comprehensive synthesis of available evidence is crucial to guide clinical practice. While previous systematic reviews have evaluated irrigation solutions for PJI prevention, to our knowledge, no Network Meta-Analysis (NMA) has been conducted to compare their efficacy in eradicating biofilms associated with established PJIs. NMAs provide a valuable framework for integrating data across multiple studies, allowing for indirect comparisons between interventions and ranking treatments based on effectiveness. This study aims to address this gap analysis in vitro results of the efficacy of various antiseptic solutions against biofilms associated with PJIs. By systematically integrating data from multiple sources, we seek to identify the most effective antiseptic strategies, providing clearer guidance for clinical decision-making and identifying areas for future research.

In summary, our research represents the first attempt to employ an NMA framework to compare antiseptic irrigation solutions for biofilm eradication in the context of PJIs. By clarifying the relative effectiveness of these solutions, this study aims to contribute to improved surgical outcomes and inform the development of optimized PJI treatment protocols.

## 2. Materials and Methods

### 2.1. Study Design

This systematic review and meta-analysis was carried out following the Preferred Reporting Items for Systematic reviews and Meta-Analyses (PRISMA) statement and the Cochrane recommendations for systematic reviews with meta-analyses [16,17].

### 2.2. Search Strategy

A systematic literature search was conducted on PubMed, Web of Science, SCIELO, and COCHRANE databases. The final search was performed on 9 January 2024, using a predefined strategy that incorporated Medical Subject Headings (MeSH) terms and keywords specifically related to antiseptic solutions for biofilm eradication in orthopedic and trauma surgery-related surfaces. The terms used included “biofilm”, “irrigation”, and “periprosthetic joint infection”. To ensure that only studies relevant to orthopedic and trauma surgery were included, articles containing the term “dental” were explicitly excluded. Additionally, a manual review of reference lists from the included articles was performed to identify additional relevant studies that might not have been captured in the initial database search.

### 2.3. Elegibility Criteria

The inclusion criteria for study selection required that the articles be in vitro studies evaluating antiseptic solutions on biofilms cultured on surfaces relevant to orthopedic and trauma surgery, such as stainless steel, titanium, or polymethylmethacrylate. The studies included had to assess bacterial species frequently implicated in PJIs, including *Staphylococcus aureus*, *Staphylococcus epidermidis*, *Pseudomonas aeruginosa*, and *Cutibacterium acnes*. Only studies that provided quantitative biofilm reduction outcomes, such as colony-forming unit (CFU) counts before and after antiseptic exposure, were considered. Furthermore, only studies investigating antiseptic solutions commonly used in clinical practice for PJI management were included.

Exclusion criteria were applied to remove studies that cultivated biofilms on non-orthopedic surfaces, including dental or other non-implant-related surfaces. Studies evaluating antiseptic solutions in combination were excluded to ensure that individual efficacy could be assessed independently. Additionally, studies focusing on mechanical or ultrasonic biofilm removal without chemical irrigation were not included. Articles reporting exposure times exceeding 5 min were excluded, as prolonged exposure is clinically impractical. Studies lacking a control group or baseline CFU quantification were also removed, along with non-original research such as reviews, case reports, and conference abstracts.

### 2.4. Study Selection

Two independent reviewers conducted the screening of titles and abstracts to identify eligible studies. Full-text articles were retrieved and assessed based on the predefined inclusion and exclusion criteria. Any disagreements between the reviewers were resolved through discussion, with the involvement of a third reviewer when necessary.

### 2.5. Data Collection

For each included study, data extraction focused on specific variables, including study characteristics (author names, year of publication), biofilm characteristics (surface type, bacterial species, and maturation time), treatment characteristics (type and concentration of antiseptic solution, exposure time), and outcome measures (CFU reduction, percentage of biofilm eradication, and statistical significance).

### 2.6. Quality Assessment

The risk of bias was assessed using the National Program for State Toxicology Tool (PNTEU) risk of bias tool, which has been adapted specifically for in vitro studies [18]. This tool evaluates several domains, including the consistency of experimental conditions across study groups, whether the exposure to antiseptic solutions was randomized, whether outcome assessment was blinded, and whether all expected outcomes were reported completely without selective omission. Each domain was categorized as “definitely low”, “probably low”, “probably high”, or “definitely high” risk of bias. While no studies were excluded based on their risk of bias, potential methodological weaknesses were considered in the final interpretation of results.

### 2.7. Statistical Analyses

An NMA was conducted to compare the efficacy of the different antiseptic solutions using a multivariate random-effects model. The log ratio of means (logRoM) was used as the effect size metric, with 95% confidence intervals (CI). Residual heterogeneity was measured using the τ^2^ statistic, where values greater than 9.76 indicated substantial heterogeneity. A likelihood ratio test was applied to assess consistency by comparing direct and indirect effects. The ranking of treatments was determined using *p*-values, where higher values indicated greater efficacy. Publication bias was evaluated using Tshwang’s rank regression method, which analyzed standardized residuals to detect asymmetry [19]. All statistical analyses were performed in R (version 4.4.1, R Foundation for Statistical Computing, Vienna, Austria) using the metafor package.

## 3. Results

### 3.1. Study Selection

**Figure 1:**
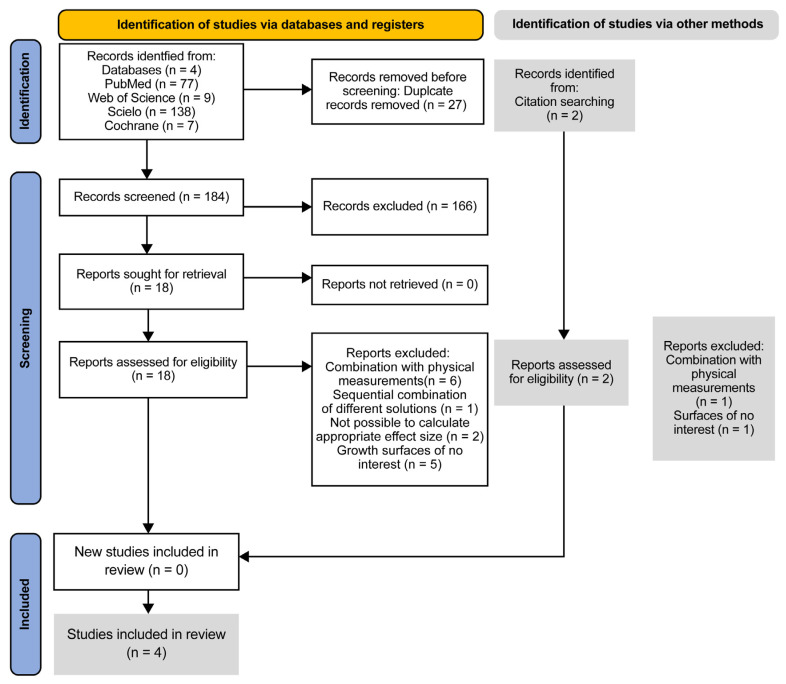
PRISMA flow diagram of included studies.

A total of 233 studies were identified through the systematic database search. After the removal of 27 duplicates, 206 unique articles were screened based on titles and abstracts. Of these, 18 studies were selected for full-text review. Following detailed assessment, 14 studies were excluded for failing to meet the eligibility criteria. The primary reasons for exclusion included the use of biofilm models on non-orthopedic surfaces (n = 6), studies evaluating mechanical biofilm removal techniques without chemical irrigation (n = 4), and studies lacking quantitative biofilm reduction data (n = 4). Ultimately, four studies met all the inclusion criteria and were included in the NMA. The study-selection process is summarized in Figure 1.

### 3.2. Characteristics of the Included Studies

The four included studies investigated a total of 10 different antiseptic solutions at varying concentrations and exposure times. These solutions were tested on mature biofilms formed on orthopedic and trauma surgery-related surfaces, including stainless steel, titanium, and polymethylmethacrylate. The bacterial species tested across the included studies comprised methicillin-sensitive *Staphylococcus aureus*, methicillin-resistant *Staphylococcus aureus*, *Staphylococcus epidermidis*, *Pseudomonas aeruginosa*, and *Cutibacterium acnes*. Exposure times ranged from 1 to 5 min, reflecting clinically relevant intraoperative application durations. CFU in all studies were obtained after sonication of the studied surfaces. The detailed characteristics of the included studies are presented in Table 1.

### 3.3. Quality Assessment

The overall risk of bias of the studies included was low. However, in none of the trials were masking techniques performed on the solutions under study. Table 2 shows the results of the analysis using the PNTEU risk of bias tool.

### 3.4. Antimicrobial Efficacy of Antiseptic Solutions

**Figure 2:**
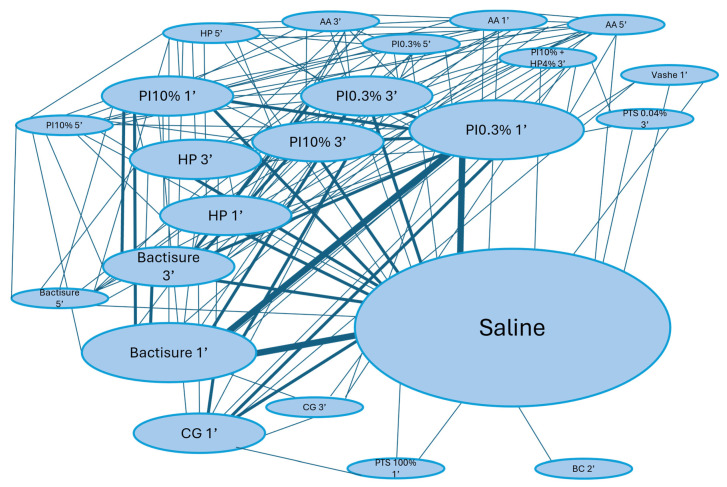
Network plot of the irrigation solutions studied. Treatments are represented by solution acronym, concentration of the solution if more than one, and time of exposure expressed in minutes. PI: Povidone Iodine, AA: Acetic acid, HP: Hydrogen peroxide, CG: Clorhexidine gluconate, PTS: Prontosan, and BC: Benzalkonium Chloride.

A total of 22 antiseptic formulations were assessed across the included studies. In all cases, isotonic saline solution was employed as a control representing 23 treatment groups (Table 3) allowing the study of 157 pairs of NMA comparisons (Figure 2). The complete NMA results are available at Appendix A.

The NMA revealed that PI at 10% concentration consistently demonstrated the highest efficacy in reducing biofilm burden, with logRoM values of −12.02 (95% CI: −14.04 to −9.99) for 5-min exposure, −11.84 (95% CI: −13.86 to −9.82) for 3-min exposure, and −11.41 (95% CI: −13.43 to −9.38) for 1-min exposure.

**Figure 3:**
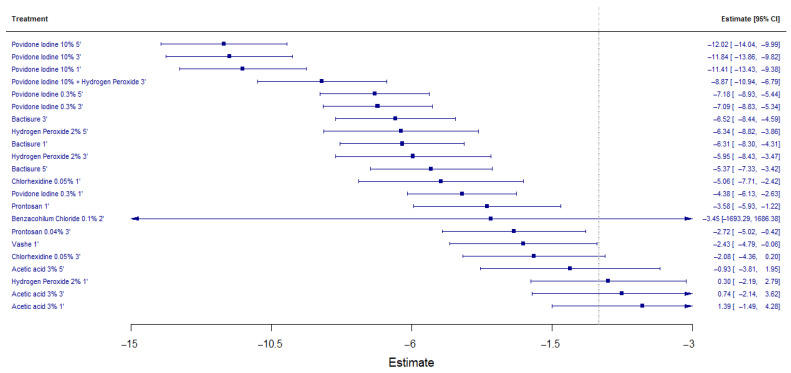
Forest plot showing the logRoM of the contrasts studied and the 95% confidence intervals with saline as reference (vertical dotted line).

Other solutions exhibiting significant antibiofilm activity included Bactisure^®^ (Zimmer-Biomet, Warsaw, IN, USA) (5-min exposure: logRoM −8.25, 95% CI: −10.47 to −6.03), chlorhexidine gluconate 0.05% (3-min exposure: logRoM −6.91, 95% CI: −9.02 to −4.80), and hydrogen peroxide 3% (5-min exposure: logRoM −6.38, 95% CI: −8.45 to −4.31). In contrast, acetic acid 3% and saline solution demonstrated the lowest efficacy in biofilm reduction. Figure 3 shows the forest plot of the estimated values of the effect measured in logRoM for all the solutions analyzed.

### 3.5. Ranking of Antiseptic Solutions

The ranking probability analysis, based on *p*-scores, confirmed that PI 10% for 5 min was the most effective antiseptic solution (*p*-score = 0.977), followed by PI 10% for 3 min (*p*-score = 0.932) and PI 10% for 1 min (*p*-score = 0.887). The combination of PI 10% with hydrogen peroxide 3% ranked fourth (*p*-score = 0.836), while PI 0.3% for 5 min completed the top five (*p*-score = 0.761).

Lower-ranking solutions included chlorhexidine gluconate 0.05% (*p*-score = 0.507), benzalkonium chloride 0.1% (*p*-score = 0.499), and acetic acid 3% for 1 min (*p*-score = 0.042), indicating limited efficacy in reducing biofilm formation. The complete ranking of all treatments is illustrated in Table 4.

### 3.6. Heterogeneity and Consistency Analysis

Residual heterogeneity across studies was assessed using the τ^2^ statistic (τ^2^ = 9.76), indicating substantial heterogeneity in treatment effects. To evaluate consistency between direct and indirect comparisons, a likelihood ratio test was performed, revealing no significant inconsistency across studies (*p* = 1.00). Consequently, the original random-effects model was retained without additional adjustments for inconsistency.

### 3.7. Publication Bias Assessment

Publication bias was analyzed using Tshwang’s rank regression method, which evaluates standardized residuals versus rank distributions. The regression analysis demonstrated an upward trend in residuals with increasing ranks, suggesting potential bias favoring studies reporting higher treatment effects. The coefficient for the rank term was 0.0182 (*p* < 0.001), indicating a statistically significant relationship. This trend was particularly evident for underrepresented antiseptic solutions, such as benzalkonium chloride, which was assessed in only one included study from over 20 years ago.

## 4. Discussion

This NMA provides the first comprehensive ranking of antiseptic solutions for biofilm eradication on orthopedic and trauma surgery-related surfaces, using a robust quantitative approach. Our findings demonstrate that PI 10% was the most effective solution, achieving a logRoM of −12.02 (95% CI: −14.04 to −9.99) at 5-min exposure, significantly outperforming all other treatments. Notably, PI 10% for 3 min also demonstrated substantial efficacy (logRoM −11.84), suggesting that shorter exposure times may still be clinically effective. The combination of PI 10% with hydrogen peroxide 3% also ranked among the top antiseptics, reinforcing the hypothesis that synergistic antiseptic strategies may enhance biofilm elimination.

The clinical implications of these findings are highly relevant to PJIs management, particularly in the context of intraoperative antiseptic irrigation protocols. Current surgical guidelines emphasize the importance of irrigation solutions in PJI management but do not specify a superior agent [15,22,23,24]. Our results provide strong evidence to support PI 10% as a first-line irrigation solution in orthopedic surgery. This study also highlights Bactisure^®^, hydrogen peroxide, and chlorhexidine as alternative antiseptics with moderate efficacy, providing potential options when PI is contraindicated.

Our study combined data from different bacteria biofilm to make our results more generalizable. However, it is worth noting that the efficacy of irrigation solutions may vary over different bacterial biofilm. O’Donnell et al. found that povidone iodine performed better on MRSA biofilms and Bactisure^®^ performed better with *P. aeruginosa*, although both reached eradication CFU count limits [14]. Márquez-Gómez et al. found that MSSA was the organism that was slightly more resistant to the solutions tested [20].

Although PI 10% demonstrated the highest antibiofilm activity, its clinical application must be balanced against potential cytotoxic effects. Studies have shown that high concentrations of PI can impair fibroblast function, delay wound healing, and induce chondrocyte apoptosis [25,26]. Given that periarticular soft tissues are often exposed to antiseptic irrigation during orthopedic procedures, optimizing the concentration and exposure time of PI is crucial to minimizing cytotoxicity while maintaining antibiofilm efficacy. Although these in vitro results are not well-proven in real situations, and chondrocytes are not present in the setting of a PJI, alternatives such as PI 0.3%, which demonstrated significant biofilm eradication with potentially lower cytotoxic risk, should be taken in consideration until more data about safety in real conditions is available [27].

From a practical standpoint, Bactisure^®^ and hydrogen peroxide remain viable alternatives, particularly in cases where PI is contraindicated. However, hydrogen peroxide produces excessive foaming, which can obscure the surgical field and complicate intraoperative visualization [28]. This highlights the need for further evaluation of intraoperative usability, as antiseptic solutions must be not only effective but also practical for real-world surgical applications.

This study represents the first NMA comparing antiseptic solutions specifically for biofilm eradication in orthopedic surgery, addressing a critical gap in the literature [29]. Our use of logRoM as a quantitative metric provides a robust and interpretable measure of antiseptic efficacy, allowing direct ranking of treatments.

However, several limitations must be acknowledged. First, this study is based entirely on in vitro experiments, which do not fully replicate in vivo conditions. While biofilm eradication was assessed via CFU reduction, this metric does not account for bacterial regrowth or host immune interactions. Nonetheless, in vitro studies provide a controlled setting to isolate the effects of antiseptic solutions on bacterial biofilms without the confounding variables present in clinical scenarios. This allows for a clearer assessment of their antimicrobial properties. In our study, we wanted to combine different variables that can be present together in the real practice, such as different surfaces and different bacteria. Additionally, while in vitro results cannot directly translate to clinical outcomes, they provide critical preliminary data that guide the selection of antiseptics for further preclinical and clinical research. Despite this, our study provides the most comprehensive synthesis of available in vitro evidence to date, offering valuable insights into the comparative efficacy of antiseptic solutions against biofilms in the context of periprosthetic joint infections. Future research should focus on bridging the gap between laboratory findings and clinical applications through well-designed in vivo and clinical studies.

Second, significant heterogeneity was detected (τ^2^ = 9.76), reflecting variability in bacterial strains, exposure times, and surface materials. Although a random-effects model was used to account for this variability, residual heterogeneity remains a concern.

Third, publication bias was detected, suggesting that studies with lower efficacy outcomes may have been underrepresented. The Tshwang’s rank regression method confirmed a significant bias coefficient (*p* < 0.001), reinforcing the need for caution in interpreting ranking probabilities.

To bridge the gap between in vitro and clinical applications, future studies should focus on in vivo models evaluating antiseptic solutions under physiological conditions. Specifically, randomized controlled trials assessing PI 10% as an irrigation solution in PJI surgeries are necessary to confirm its clinical efficacy and safety.

Further investigations should also explore synergistic antiseptic strategies. The combination of PI 10% with hydrogen peroxide 3% demonstrated strong efficacy, suggesting potential benefits from dual-agent irrigation approaches. In this direction, it has already been studied the sequential use of different irrigation solutions that may bring some benefits [20]. Additional studies are needed to determine whether lower concentrations of these solutions could achieve comparable efficacy while minimizing toxicity.

Finally, standardized antiseptic testing protocols are needed. The lack of uniformity in exposure times, bacterial strains, and biofilm models as well as the report of results limits the comparability of studies. Establishing guidelines for antiseptic testing could improve reproducibility and facilitate more meaningful comparisons across studies.

## 5. Conclusions

To conclude, the aim of this NMA was to know which antiseptic irrigation solutions is more effective against bacterial biofilm. Our study provides strong evidence that PI 10% is the most effective antiseptic solution for biofilm eradication on orthopedic implant surfaces. The findings support PI 10% as a leading candidate for intraoperative irrigation in PJI surgeries, although cytotoxicity remains a key consideration. Alternative solutions, with better safety condition, including further diluted povidone iodine, Bactisure^®^, chlorhexidine, and hydrogen peroxide, demonstrated moderate efficacy but require further clinical validation. Future studies should focus on in vivo testing, optimizing antiseptic concentrations, and evaluating combination strategies to enhance biofilm eradication while minimizing adverse effects.

## Figures and Tables

**Table 1:** Main characteristics of included studies.

Study	Márquez-Gómez (2023) [20]	Premkumar (2021) [13]	O’Donnell (2021) [14]	Moussa (1996) [21]
**Surfaces**	Stainless Steel	Ti6Al4V *; PMMA **^†^**; Polystyrene	Titanium disc	Stainless Steel
**Irrigation solutions**	PI 0.3%; PI 10%; H_2_O_2_ 3%; AA 3%; Bactisure; saline	PI 10%; PI 0.3%; GCH 0.05%; PI10% + H_2_O_2_ 3%; Prontosan 0.04%; Bactisure; saline	Bactisure; CG 0.05%; PI 0.35%; Vashe; Prontosan; saline	Benzalkonium Chloride 0.1%; saline
**Exposure times**	1′; 3′; 5′	1′; 3′	1′	1′; 5′
**Bacteria species**	MSSA ^‡^; MRSA **^§^**; *S. epidermidis; P. aeuriginosa*	MSSA ^‡^	MSSA ^‡^; *S. epidermidis*; *P. aeruginosa*; *C. acnes*	*P. aeruginosa*; *S. epidermidis*; MSSA **^‡^**
**Biofilm maturation time**	24 h	72 h	72 h	24 h

This table represents the growing surfaces, irrigation solutions, bacteria species, and maturation times of biofilm for each study. Irrigation solutions are represented by their abbreviation and concentration. PI: Povidone Iodine, H_2_O_2_: Hydrogen peroxide, AA: Acetic Acid, CG: Chlorhexidine gluconate. * Ti6Al4V: titanium alloy from an acetabular component. ^†^ PMMA: Polymethyl methacrylate. ^‡^ MSSA: Methicillin-sensitive *Staphylococcus aureus*. ^§^ MRSA: Methicillin-resistant *Staphylococcus aureus*.

**Table 2:** Results of the risk of bias analysis according to the National Program for State Toxicology Tool (PNTEU). D1: Screening; D2: Execution; D3: Atriction; D4: Detection; D5: Selective reporting; D6: Other.

Studies	D1	D2	D3	D4	D5	D6
Márquez-Gómez 2023 [20]						
O’Donnell 2021 [14]						
Premkumar 2021 [13]						
Moussa 1996 [21]						

Judgement: 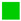 Definitely low; 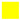 Probably low; 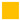 Probably high; 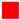 Definitely high.

**Table 3:** Treatment groups included in the Network Meta-Analysis. Groups are represented by the name of the solution, concentration expressed in percentage, and duration of exposure in min.

Antiseptic Solutions, Concentration, and Exposure Times
Acetic Acid 3% 1′
Acetic Acid 3% 3′
Acetic Acid 3% 5′
Benzalkonium Chloride 1:1000 2′
Bactisure 1′
Bactisure 3′
Bactisure 5′
Chlorhexidine Gluconate 0.05% 1′
Chlorhexidine Gluconate 0.05% 3′
Hydrogen Peroxide 1′
Hydrogen Peroxide 3′
Hydrogen Peroxide 5′
Prontosan 0.04% 3′
Povidone Iodine 0.3% 1′
Povidone Iodine 0.3% 3′
Povidone Iodine 0.3% 5′
Povidone Iodine 10% 1′
Povidone Iodine 10% 3′
Povidone Iodine 10% 5′
Povidone Iodine 10% + Hydrogen Peroxide 4% 3′
Prontosan 1′
Vashe 1′
Saline Solution

**Table 4:** Ranking of treatments based on *p*-scores.

Irrigation Solutions Ranking	*p*-Score
Povidone Iodine 10% 5′	0.977
Povidone Iodine 10% 3′	0.932
Povidone Iodine 10% 1′	0.887
Povidone Iodine 10% + Hydrogen Peroxide 4% 3′	0.836
Povidone Iodine 0.3% 5′	0.761
Povidone Iodine 0.3% 3′	0.712
Bactisure 5′	0.673
Hydrogen Peroxide 5′	0.655
Bactisure 3′	0.637
Hydrogen Peroxide 3′	0.581
Bactisure 1′	0.519
Chlorhexidine Gluconate 0.05% 1′	0.507
Benzalkonium Chloride 0.1% 2′	0.499
Povidone Iodine 0.3% 1′	0.437
Prontosan 1′	0.381
Prontosan 0.04% 3′	0.321
Vashe 1′	0.298
Chlorhexidine Gluconate 0.05% 3′	0.276
Acetic Acid 3% 5′	0.213
Saline	0.134
Hydrogen Peroxide 1′	0.120
Acetic Acid 3% 3′	0.103
Acetic Acid 3% 1′	0.042

## Data Availability

Complete results of the Network Meta-Analysis are available at Appendix A.

## Appendix A

### Multivariate Meta-Analysis Model (k = 157; Method: REML)

Variance Components:

outer factor: study (nlvls = 4)

inner factor: comp (nlvls = 43)

	**Estim**	**Sqrt**	**Fixed**
tau^2	97.596	31.240	no
rho	0.5000		yes

Test for Residual Heterogeneity:

QE(df = 135) = 4269.5104, *p*-val < 0.0001

Test of Moderators (coefficients 1:22):

QM(df = 22) = 1805.2799, *p*-val < 0.0001

Model Results:

	**estimate**	**se**	**zval**	**pval**	**ci.lb**	**ci.ub**	**sig.**
AA31	13.917	14.720	0.9454	0.3444	−14.934	42.768	
AA33	0.7431	14.697	0.5056	0.6131	−21.375	36.237	
AA35	−0.9278	14.687	−0.6317	0.5276	−38.064	19.509	
BA102	−34.528	8621.773	−0.0040	0.9968	−16,932.894	16,863.837	
Bactisure1	−63.063	10.168	−62.024	<0.0001	−82.992	−43.135	***
Bactisure3	−65.177	0.9824	−66.347	<0.0001	−84.431	−45.923	***
Bactisure5	−53.722	0.9967	−53.900	<0.0001	−73.257	−34.187	***
CHG0051	−50.647	13.482	−37.566	0.0002	−77.072	−24.222	***
CHG0053	−20.837	11.635	−17.909	0.0733	−43.641	0.1967	.
H2O21	0.3002	12.690	0.2366	0.8130	−21.869	27.873	
H2O23	−59.514	12.666	−46.988	<0.0001	−84.339	−34.690	***
H2O25	−63.402	12.661	−50.077	<0.0001	−88.217	−38.587	***
PhmbBtn3	−27.161	11.730	−23.154	0.0206	−50.152	−0.4169	*
PI031	−43.825	0.8929	−49.082	<0.0001	−61.326	−26.325	***
PI033	−70.872	0.8910	−79.540	<0.0001	−88.335	−53.408	***
PI035	−71.843	0.8916	−80.578	<0.0001	−89.319	−54.368	***
PI101	−114.091	10.336	−110.384	<0.0001	−134.349	−93.833	***
PI103	−118.400	10.331	−114.602	<0.0001	−138.649	−98.150	***
PI105	−120.152	10.336	−116.252	<0.0001	−140.409	−99.895	***
PI10PO3	−88.694	10.586	−83.781	<0.0001	−109.443	−67.945	***
Prontosan1	−35.762	12.018	−29.757	0.0029	−59.317	−12.208	**
Vashe1	−24.253	12.044	−20.136	0.0441	−47.859	−0.0646	*
Signif. codes: 0 ‘***’ 0.001 ‘**’ 0.01 ‘*’ 0.05 ‘.’ 0.1 ‘ ’.							
